# Baicalin ameliorates polycystic ovary syndrome through AMP-activated protein kinase

**DOI:** 10.1186/s13048-019-0585-2

**Published:** 2019-11-13

**Authors:** Wei Wang, Jiahua Zheng, Na Cui, Lei Jiang, Han Zhou, Dan Zhang, Guimin Hao

**Affiliations:** Department of Reproduction, The Second Hospital of Hebei Medical University, Shijiazhuang, 050000 Hebei China

**Keywords:** Polycystic ovary syndrome, Baicalin, AMP-activated protein kinase, Endocrine and reproductive dysfunction, Insulin resistance

## Abstract

Polycystic ovary syndrome (PCOS) is a complex endocrine disorder and regarded as the leading cause of anovulatory infertility. PCOS is characterized by reproductive dysfunction and metabolic disorders. Baicalin (BAL) is one of the most potent bioactive flavonoids isolated from the radix of Scutellaria baicalensis. In the present study, we investigated the potential effects of BAL on PCOS in dehydroepiandrosterone-treated rats. We found that BAL notably reduced the serum levels of free testosterone, total testosterone, follicle-stimulating hormone, luteinizing hormone, progesterone, and estradiol in PCOS rats. The increase of serum insulin level and HOMA-IR was markedly inhibited by BAL. Moreover, BAL decreased body weights, increased the number of rats with the regular estrous cycle, and ameliorated ovarian histological changes and follicular development in the DHEA-treated PCOS rats. The increase of pro-inflammatory cytokines (TNFα, IL-1β, and IL-18) and decrease of anti-inflammatory cytokine (IL-10) in PCOS rats were suppressed by BAL. BAL induced a significant decrease in the mRNA expression of steroidogenic enzymes, including 3β-HSD, CYP11A1, CYP19A1, StAR, in ovarian tissues in PCOS rats. Furthermore, BAL inhibited the decrease of AMPK protein level and phosphorylation, the decrease of Akt phosphorylation and the increase of 5α-reductase enzyme 1 expression in ovarian tissues in PCOS rats. The effects of BAL were inhibited by an inhibitor of AMPK, dorsomorphin. The upregulation of AMPK contributed to the beneficial effects of BAL. The results highlight the potential role of BAL for the intervention of PCOS.

## Introduction

Polycystic ovary syndrome (PCOS) is a complex endocrine disorder and regarded as the leading cause of anovulatory infertility that affects 8–12% of reproductive-aged women [1, 2]. PCOS causes a great burden on the health care system in the whole world. For example, in the USA in 2004, it costs $4.36 billion to treat PCOS-related reproductive dysfunction and metabolic disorders [3]. PCOS is characterized by anovulation, clinical or biological hyperandrogenism and abnormalities of the ovary [4]. In addition, insulin resistance is a hallmark of PCOS [5] and is strongly associated with the reproductive and cardiometabolic complications of PCOS [6]. It is shown that nearly 40–60% of PCOS patients are overweight or obese, which promotes insulin resistance and its related metabolic and reproductive abnormalities [7]. To date, there is no ideal pharmacological intervention for PCOS. The available therapeutic measurements primarily focus on addressing reproductive dysfunction and insulin resistance [8]. Effective treatments are needed to improve health outcomes for women with PCOS.

Scutellaria baicalensis Georgi (known as huang qin) is a traditional Chinese herb, that has been shown to exhibit antioxidant, anti-inflammatory, anti-bacterial and anti-viral, antidiabetic and anti-dyslipidemic activities [9, 10]. Baicalin (BAL) is one of the most potent flavonoids isolated from the radix of Scutellaria baicalensis [11, 12]. Anti-inflammatory activity is a primary biological potential of BAL in a variety of diseases and disorders [13], including rheumatoid arthritis, obesity, type 2 diabetes, respiratory diseases, inflammatory bowel diseases, cardiovascular diseases, autoimmune encephalomyelitis, etc. In particular, various studies have demonstrated that BAL could attenuate metabolic disorders in the context of obesity, insulin resistance and diabetes [14]. However, whether BAL could protect against PCOS is not known.

In the current study, we aimed to evaluate the effects of BAL on the dysregulation of hormones, histological changes of the ovary, and metabolic disorder in PCOS rats. Previous literature and our preliminary studies have suggested that activation of AMP-activated protein kinase (AMPK) is associated with the beneficial effects of BAL [15]. Thus, we examined the potential involvement of AMPK in BAL-induced effects on PCOS rats.

## Materials and methods

### Drugs and reagents

Baicalin and dehydroepiandrosterone (DHEA) were purchased from Sigma-Aldrich, USA. Antibodies against AMPK, P-AMPK, Akt, and P-Akt were acquired from Cell Signaling Technology Inc., USA. Antibodies against 5α-R1 and β-actin from Santa Cruz Biotechnology, USA. RIPA lysis from Beotime Corporation, Beijing, China. BCA TM protein assay kit from Pierce Chemical Company, Rockford, USA.

### Animals and treatment

All animal experiments were approved by the Institutional Animal Care Committee of The Second Hospital of Hebei Medical University and in strict accordance with the Guidelines for the Care and Use of Laboratory Animals (National Research Council of People’s Republic of China, 2010). Forty Wistar female rats (50–70 g, 21 days old) were purchased from the Animal Centre of Hebei Medical University. The animals were housed under a temperature-controlled condition (22 ± 2 °C), with a 12 h/12 h light/dark cycle and had free access to water.

**Fig. 1:**
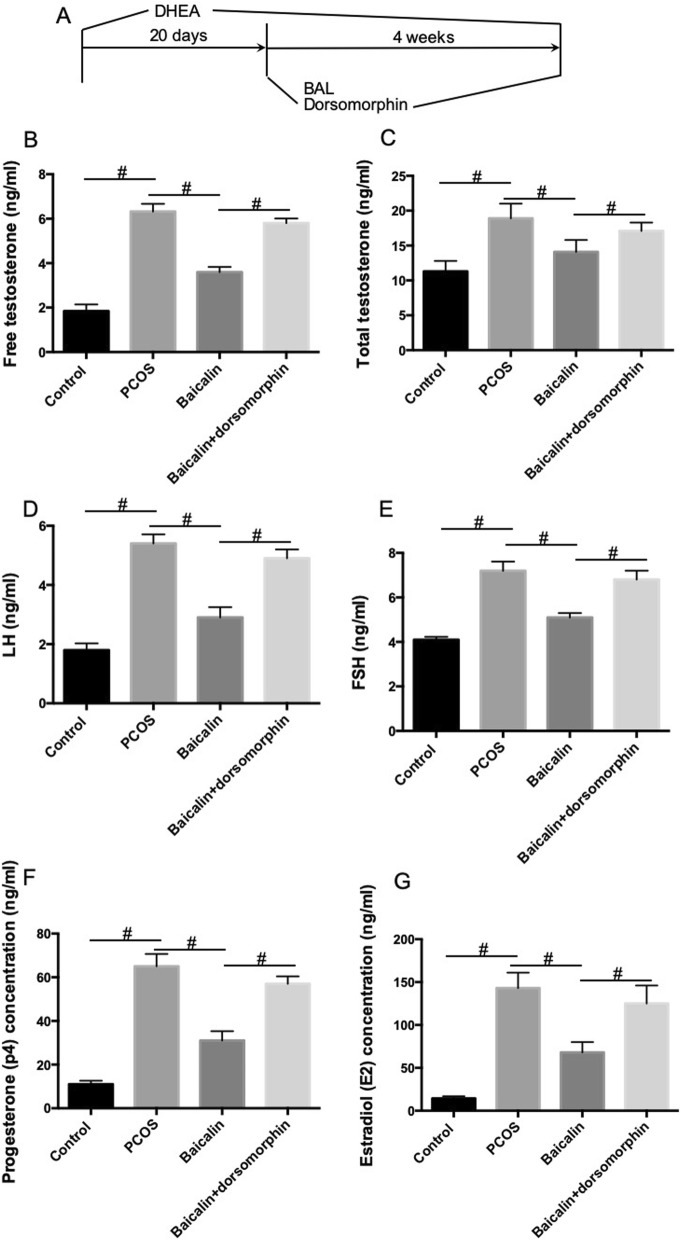
Effect of BAL on serum hormone levels in DHEA-treated PCOS rat. **a** Flowchart of the study. **b** Serum levels of free testosterone. **c** Serum levels of total testosterone. **d** Serum levels of LH. **e** Serum levels of FSH. **f** Serum levels of progesterone. **g** Serum levels of estradiol. #*P* < 0.05, statistical difference between the two groups

Rats were randomly divided into 4 groups (10 rats in each group): Control group, PCOS group, BAL group (50 mg/kg in PCOS rats), BAL + dorsomorphin group (50 mg/kg BAL+ 10 mg/kg dorsomorphin in PCOS rats). Rats in the PCOS, BAL, and BAL + dorsomorphin groups were subcutaneously injected with DHEA (Sinopharm Chemical Reagent, Co., Ltd., 6 mg/100 g·d/0.2 ml sesame oil) for 20 consecutive days to induce PCOS model with excessive androgen as previously described [16, 17]. After that, rats in the PCOS, BAL, and BAL + dorsomorphin groups were administrated continuously with DHEA for the next 4 weeks. Rats in the control group were given equal volume of sesame oil. In the following weeks, rats in the BAL and BAL + dorsomorphin groups were injected with 50 mg/kg/day BAL. Rats in the control and PCOS groups were given an equal volume of vehicle (DMSO in saline). Rats in the BAL + dorsomorphin group were also injected with 10 mg/kg/day dorsomorphin in the following 4 weeks. Rats in the control, PCOS and BAL groups were given an equal volume of vehicle (DMSO in saline). A flowchart of the study was shown in Fig. 1a.

### Determination of serum levels of hormones and metabolic variables

After the treatment, rats were fasted overnight and blood was centrifuged at 3000 rpm for 10 min to collect serum. Serum levels of insulin, follicle-stimulating hormone (FSH), luteinizing hormone (LH), estradiol (E2), progesterone (P) were detected using ELISA kits (Uscn Life Science, Inc., Wuhan, China). Free testosterone and total testosterone levels were measured using ELISA kits (Sigma-Aldrich, USA). These assays were carried out according to the manufacturer’s instructions. The homeostasis model assessment of insulin resistance index (HOMA-IR) was calculated according to the following formula: plasma glucose (mmol/l) x serum insulin (mIU/l)/22.5.

### Determination of pro-inflammatory factors

After the sacrifice of the rats, ovarian tissues were collected followed by the removal of coagulation and appendage. 10% ovarian tissue homogenates in precooled saline were centrifuged at 3000 rpm for 10 min to collect the supernatant. Levels of TNFα, IL-1β, IL-10, and IL-18 in ovarian tissue homogenates were measured using ELISA kits (R&D Systems, USA).

### Hematoxylin-eosin (HE) staining and histological changes

Ovarian tissue was immediately cut, fixed and embedded with paraffin. The embedded tissue was cut into 5-μm sections, followed by classical HE staining procedure. Pathological changes were observed under an optical microscope (Olympus, Japan).

The percentage of different follicles per ovary section was counted. Follicles were classified as cystic follicles, primary follicles, early antral follicles, and atretic follicles according to the previous study [18].

### Bodyweight and estrous cycle

Bodyweight was monitored daily throughout the experimental procedure. The estrous cycle was measured through the microscopic determination of cell types on vaginal smears.

### Western blotting analysis

Fifty milligrams of ovarian tissues were homogenized in RIPA lysis buffer followed by centrifuging at 12000×g for 10 min at 4 °C and collection of supernatant. The protein concentration was determined by the BCA protein assay (Thermo Scientific, USA). An equal volume of supernatant was mixed with 2 × SDS buffer. Mixtures were separated by electrophoresis via 10% sodium dodecylsulfate-polyacrylamide gel electrophoresis (SDS-PAGE). The separated protein was transferred to a nitrocellulose (NC) membrane (Bio-Rad Instruments, CA, USA). Blocking of membranes was conducted using incubation with 5% fat-free milk at 37 °C for 1 h. Then the membranes were incubated with indicated antibodies at 4 °C overnight. After 4-time washing using TBST, membranes were incubated with goat anti-rabbit-HRP secondary antibody (Thermo Scientific, USA) at room temperature for 1 h. β-actin served as an internal control. The immunoreactive bands were visualized by chemiluminescence and quantified by densitometry using a Quantity One Analysis Software (Bio-Rad).

### RNA extraction and quantitative real-time polymerase chain reaction

One hundred milligrams of frozen ovarian tissues were homogenized using Trizol to extract total RNA. The RNA concentration was detected using a NanoDrop ND-2000 and RNA quantification was performed by spectrophotometric analysis of OD values at 260/280 nm. RNA was reversely transcribed into cDNA using RT reagent Kit with gDNA Eraser (Takara, Dalian, China) according to the manufacturer’s protocols. Quantitative RT-PCR detection of target genes was conducted using SYBR Green Master Mix (Takara, Dalian, China) on a Bio-Rad system. GAPDH was used as an internal reference. Amplification condition: an initial denaturation at 95 °C for 10 min; 95 °C for 15 s, 62 °C for 60 s, 40 cycles.

### Statistical analysis

Data were expressed as means ± standard deviation (SD). GraphPad Prism software was used to perform statistical analysis. The power analysis of the study was performed before the experiment. Enough numbers of animals were included for all the determinations. Statistical significance was evaluated using one-way analysis of variance followed by Student-Newman-Keuls test. For statistical analysis of the estrous cycle, χ^2^-tests (percentage analysis) was used which was followed by the non-parametric Kruskal–Wallis test for comparison of two groups. *P* < 0.05 was considered as the limit for statistical significance.

## Results

### BAL improves the abnormalities of serum hormone levels in DHEA-treated PCOS rats

The disorder of hormone secretion was a feature of PCOS. In the current study, we showed that serum levels of free testosterone (B), total testosterone (C), LH (D), FSH (E), progesterone (F), and estradiol (G) were significantly increased in PCOS rats (Fig. 1). As expected, the administration of BAL notably reduced the serum levels of free testosterone (B), total testosterone (C), LH (D), FSH (E), progesterone (F), and estradiol (F) in PCOS rats (Fig. 1). To validate the possible role of AMPK in BAL-exhibited effects, BAL-treated rats were injected with dorsomorphin, an inhibitor of AMPK. The results showed that BAL-induced decrease of free testosterone (B), total testosterone (C), LH (D), FSH (E), progesterone (F), and estradiol (G) levels in serum was inhibited by dorsomorphin in PCOS rats (Fig. 1). The results suggested that BAL could protect against the abnormal secretion of hormones in PCOS and the activation of AMPK may contribute to this effect of BAL.

### BAL improves insulin resistance in DHEA-treated PCOS rats

**Fig. 2:**
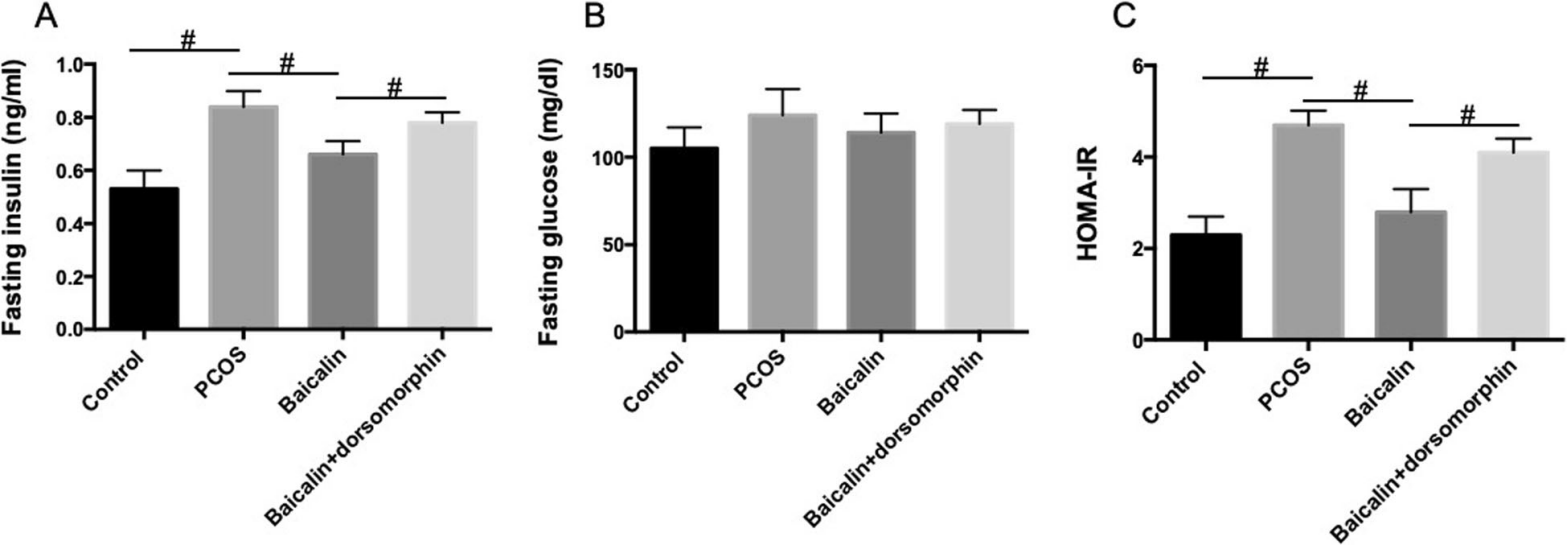
Effect of BAL on metabolic variables in DHEA-treated PCOS rat. **a** Serum levels of fasting insulin. **b** Serum levels of fasting glucose. **c** HOMA-IR. #*P* < 0.05, statistical difference between the two groups

Insulin resistance is a feature of PCOS. We examined the effect of BAL on insulin resistance in PCOS rats. As shown in Fig. 2a, the level of serum insulin was higher in PCOS rats than that in control rats. The treatment of BAL significantly decreased serum level of insulin in PCOS rats (Fig. 2a). The injection of dorsomorphin notably inhibited the effect of BAL on serum levels of insulin (Fig. 2a). Although with no statistical significance, there was a slight decrease in blood glucose level in BAL-treated rats, compared with control rats (Fig. 2b). Moreover, the HOMA-IR index was calculated to evaluate the state of insulin resistance in rats. As shown in Fig. 2c, there was a significant increase in HOMA-IR in PCOS rats, which was significantly inhibited by BAL. In addition, the BAL-induced decrease of HOMA-IR was markedly inhibited by the inhibitor of AMPK (Fig. 2c). The results indicated that BAL could attenuate insulin resistance in PCOS rats and AMPK was involved in the protective effects of BAL.

### BAL decreases body weight, improves estrous cycle and ameliorates ovarian histological changes and follicular development in DHEA-treated PCOS rat

**Fig. 3:**
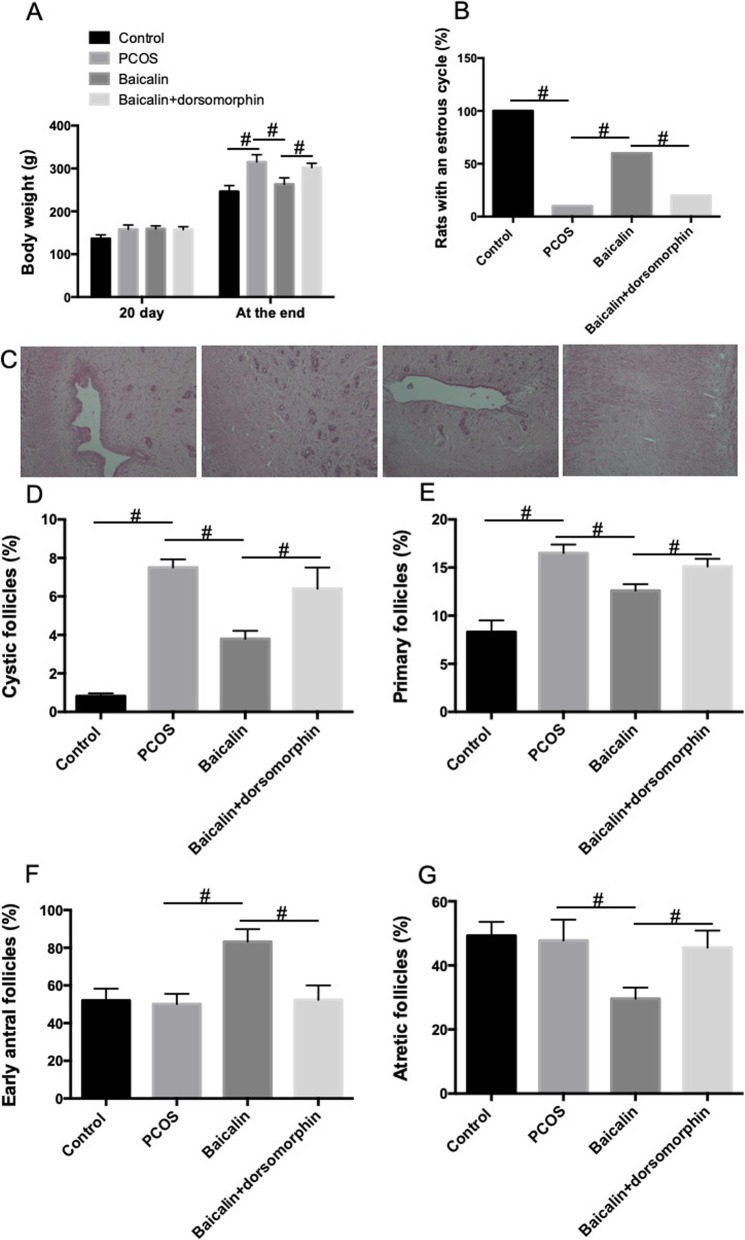
Effect of BAL on body weights, estrus cycle, histological changes and follicular development in DHEA-treated PCOS rat. **a** Body weights. **b** Estrus cycle. **c** HE staining of ovarian sections. **d** Cystic follicles. **e** Primary follicles. **f** Early antral follicles. **g** Atretic follicles. #*P* < 0.05, statistical difference between the two groups

The bodyweights of rats in PCOS group were significantly higher than those in control group (Fig. 3a). BAL decreased the body weights in DHEA-treated rats which effect was inhibited by AMPK inhibitor (Fig. 3a). The estrous cycle was used as another index of ovarian function in PCOS rats. All rats in control group exhibited a normal estrous cycle. However, DHEA-induced PCOS rats showed a significantly impaired cycle; only 1 of 10 rats exhibited a normal estrous cycle in PCOS group (Fig. 3b). In BAL group, 6 of 10 rats showed a normal estrous cycle (Fig. 3b). In the presence of dorsomorphin, the number of rats with a normal estrous cycle reduced to 2 of 10 rats in BAL+ dorsomorphin group (Fig. 3b).

We also detected the effect of BAL on histological changes and follicular development in ovarian tissues in PCOS rats. We observed corpus luteum and follicles in control rats and there were oocytes in nearly mature follicles and fewer follicular cell layers (Fig. 3c). In DHEA-treated rats, we observed multiple primary follicles and few follicles and no oocytes in nearly mature follicles (Fig. 3c). The follicular cell layer was thicker and granulosa cells were arranged loosely (Fig. 3c). Compared with rats in PCOS group, BAL ameliorated histopathological changes in ovarian tissue, as reflected by multiple corpus luteum and follicles, oocytes in nearly mature follicles, tightly arranged granulosa cells and thin ovarian cell layer (Fig. 3c). In contrast, BAL-induced attenuation of histopathological changes was inhibited by dorsomorphin (Fig. 3c).

Moreover, we also evaluated the effect of BAL on the percentage of different follicles per ovary section. We showed that the percentages of cystic follicles and primary follicles were significantly increased in PCOS rats (Fig. 3d and e). BAL administration significantly decreased the percentages of cystic follicles and primary follicles, which effect was inhibited by dorsomorphin (Fig. 3d and e). Although there was no significant changes of the percentages of early antral follicles and atretic follicles between PCOS and control rats, BAL significantly increased the percentage of early antral follicles and decreased the percentage of atretic follicles (Fig. 3f and g). The effect of BAL on early antral follicles and atretic follicles was inhibited by dorsomorphin (Fig. 3f and g). The results demonstrated that BAL ameliorated ovarian functional and histological changes and follicular development in DHEA-treated PCOS rats and AMPK was involved in this effect.

### BAL ameliorates inflammation in ovarian tissues in DHEA-treated PCOS rat

**Fig. 4:**
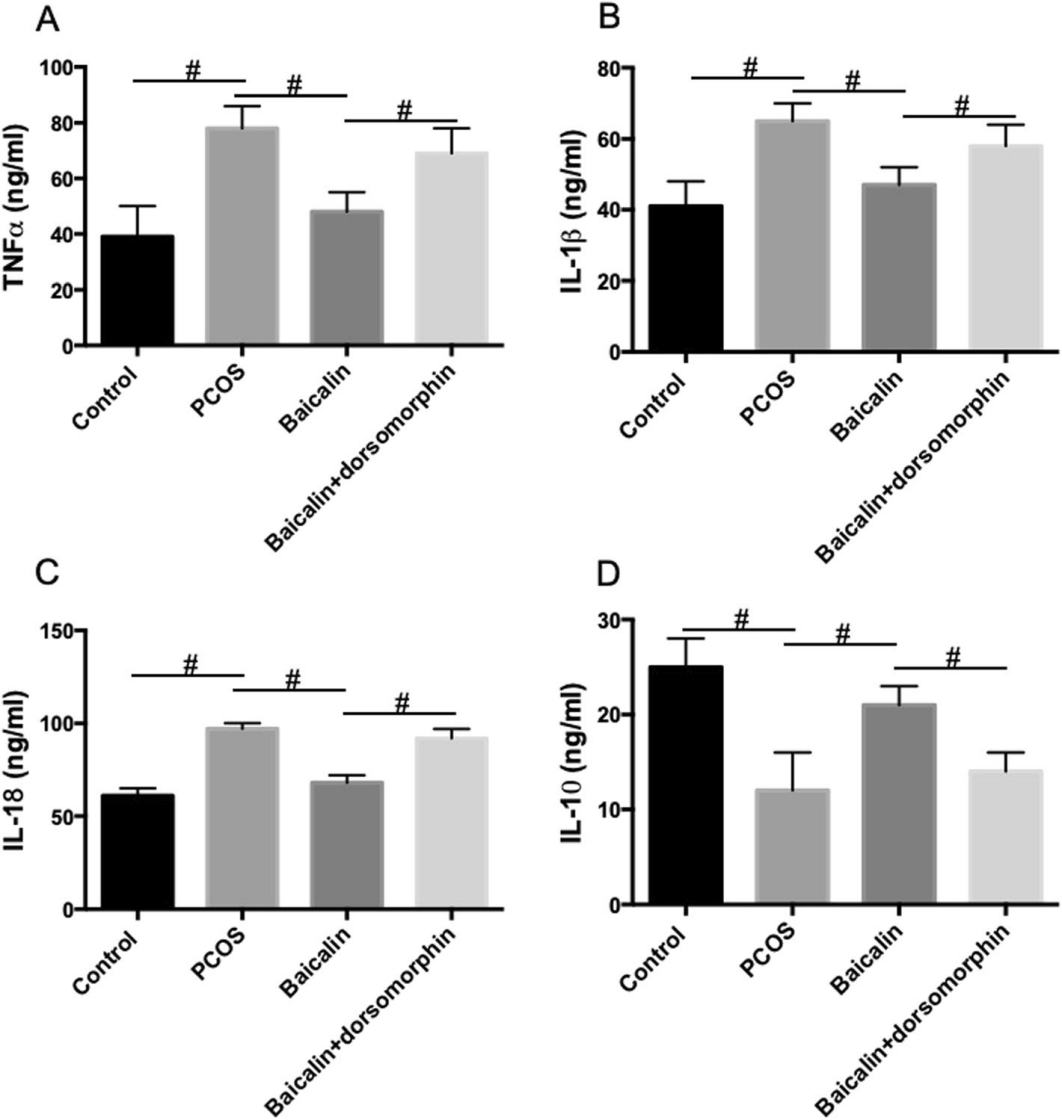
Effect of BAL on inflammation in DHEA-treated PCOS rat. **a** Ovarian levels of TNFα. **b** Ovarian levels of IL-1β. **c** Ovarian levels of IL-18. **d** Ovarian levels of IL-10. #*P* < 0.05, statistical difference between the two groups

Inflammation is considered to be associated with the pathological outcomes of PCOS. We also evaluated the effect of BAL on the content of pro-inflammatory and anti-inflammatory cytokines. As shown in Fig. 4, the levels of TNFα (A), IL-1β (B), and IL-18 (C) in ovarian tissues of PCOS rats were significantly increased, while the level of IL-10 (D) was decreased. BAL inhibited the increase of TNFα (A), IL-1β (B), and IL-18 levels and the decrease of IL-10 level in ovarian tissues in PCOS rats Fig. 4. Dorsomorphin significantly inhibited the effect of BAL on these pro-inflammatory and anti-inflammatory cytokines (Fig. 4). The results indicated that BAL ameliorated inflammation in ovarian tissues in DHEA-treated PCOS rat via an AMPK-dependent manner.

### BAL decreases the mRNA expression of steroidogenic enzymes in ovarian tissues in DHEA-treated PCOS rat

**Fig. 5:**
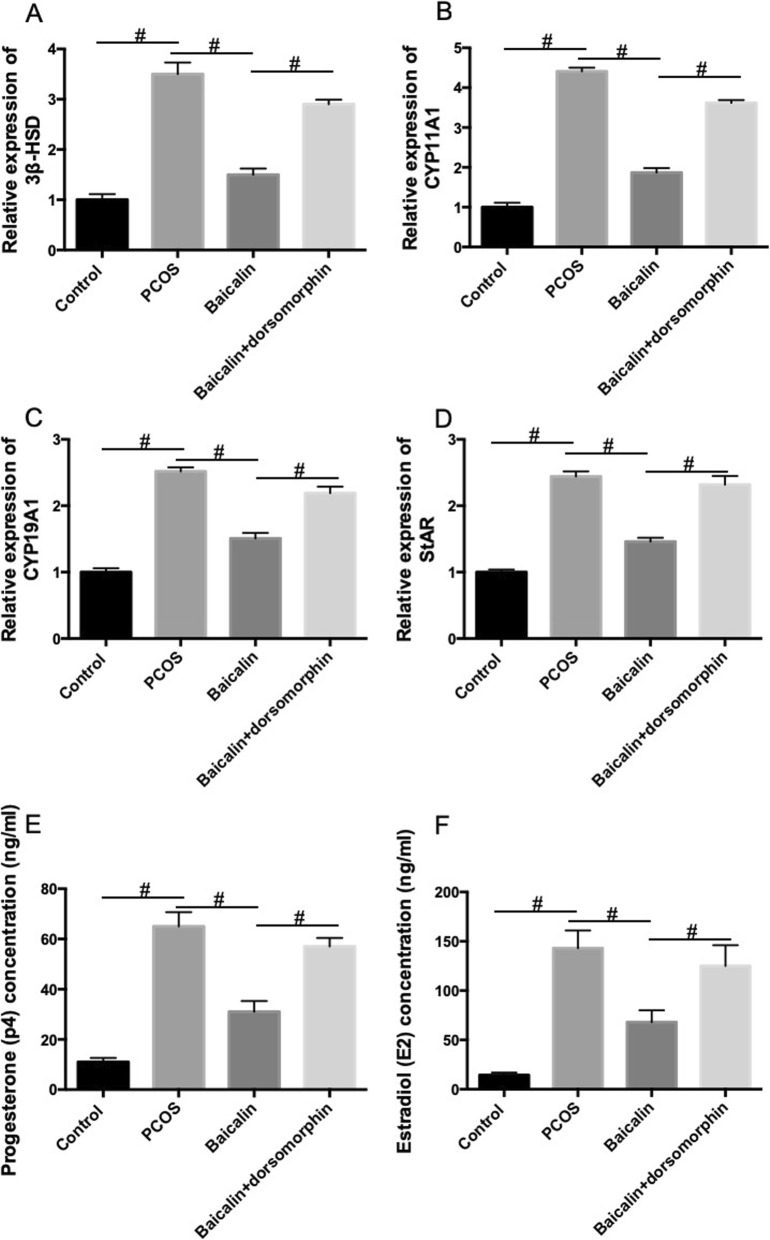
Effect of BAL on steroidogenic enzymes mRNA expression in DHEA-treated PCOS rat. **a** mRNA expression of 3β-HSD. **b** mRNA expression of CYP11A1. **c** mRNA expression of CYP19A1. **d** mRNA expression of StAR. **e** Progesterone concentration. **f** Estradiol concentration. #*P* < 0.05, statistical difference between the two groups

The effect of BAL on the mRNA expression of steroidogenic enzymes in ovarian tissues was also determined. In Fig. 5, we showed a significant increase of the mRNA expression of steroidogenic enzymes, including 3β-HSD (A), CYP11A1 (B), CYP19A1 (C), StAR (D), in ovarian tissues in DHEA-treated PCOS rat. The increase of these steroidogenic enzymes mRNA expression was notably inhibited by BAL (Fig. 5). Moreover, the inhibitory effect of BAL on the mRNA expression of steroidogenic enzymes was blocked by dorsomorphin (Fig. 5). Progesterone and estradiol concentrations were decreased by BAL in PCOS rats, which effect was inhibited by AMPK inhibitor (Fig. 5e and f). The results suggested that BAL exhibited an inhibitory effect on the mRNA expression of steroidogenic enzymes in ovarian tissues in PCOS, in which process the activation of AMPK was involved.

### BAL activated AMPK and Akt, but inhibited 5α-R1 in ovarian tissues in DHEA-treated PCOS rat

**Fig. 6:**
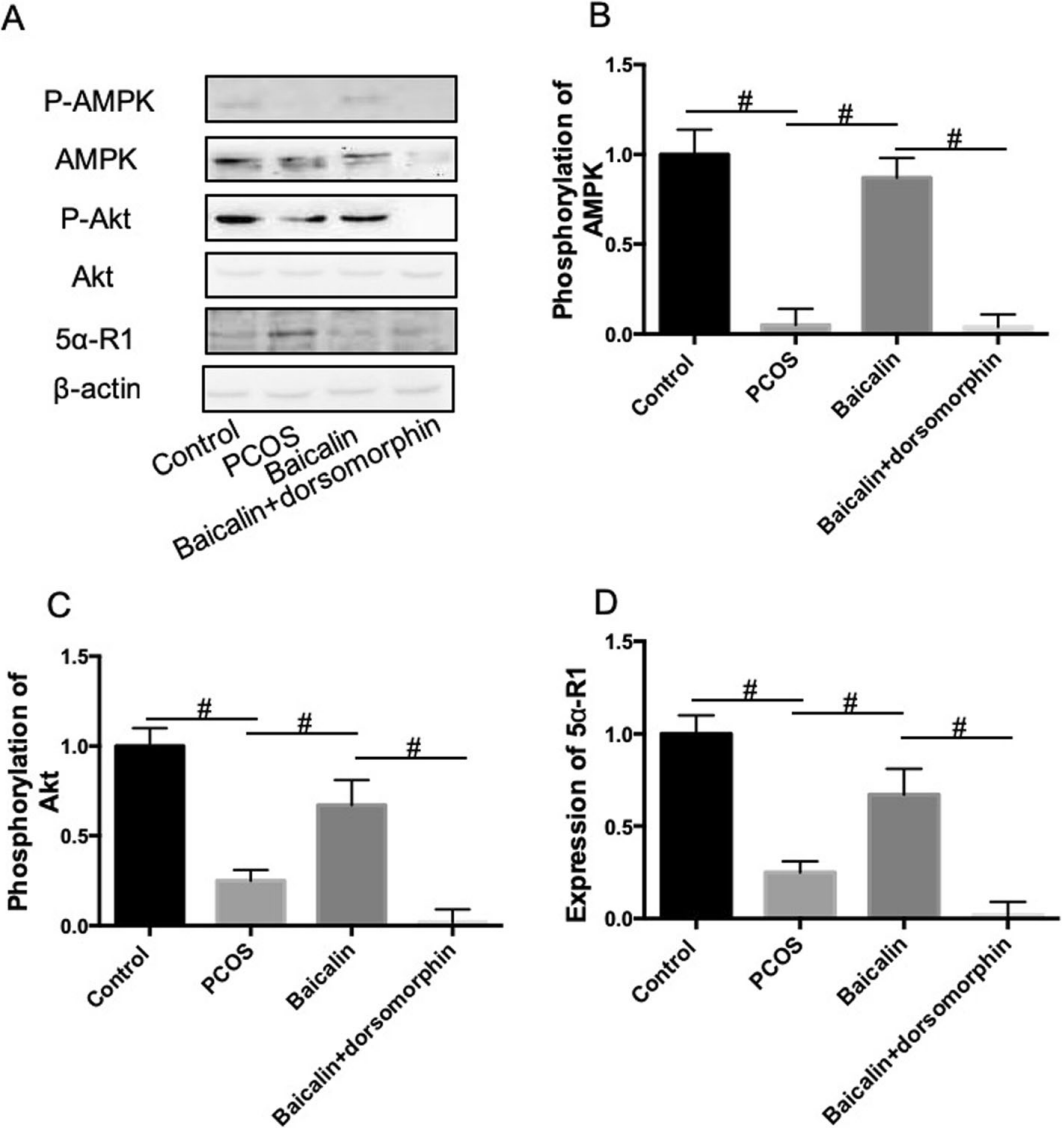
Effect of BAL on AMPK, Akt, and 5α-R1 in DHEA-treated PCOS rat. **a** Protein expression of total-AMPK, P-AMPK, total-Akt, P-Akt, and 5α-R1. **b** Quantitative analysis of the phosphorylation of AMPK. **c** Quantitative analysis of the phosphorylation of Akt. **d** Quantitative analysis of the expression of 5α-R1. #*P* < 0.05, statistical difference between the two groups

We next tried to explore the mechanism of BAL-exhibited effect on PCOS. The activation of AMPK by BAL was confirmed by protein expression of total and phosphorylation of AMPK (Fig. 6a and b). Dorsomorphin injection significantly decreased AMPK expression and phosphorylation in BAL-treated PCOS rats (Fig. 6a and b). We also examined the changes of Akt expression and phosphorylation. Phosphorylation of Akt was significantly decreased under PCOS condition, while BAL notably increased Akt phosphorylation in ovarian tissues, which effect was inhibited AMPK inhibitor (Fig. 6a and c). The results indicated that BAL upregulated Akt signaling through AMPK activation. We also determined the expression of 5α-reductase type 1(5α-R1). 5α-R1 protein expression was increased in ovarian tissues of PCOS rats, and decreased by BAL treatment (Fig. 6a and d). Furthermore, this effect was inhibited by dorsomorphin (Fig. 6a and d), indicating that BAL inhibited 5α-R1 expression which was dependent on AMPK signaling.

## Discussion

PCOS is a complex syndrome that is associated with abnormal secretion, insulin resistance, and inflammation. Importantly, hyperinsulinemia and insulin resistance are critical hallmarks and contributors to the progression of metabolic and reproductive dysfunction in women with PCOS. Hyperinsulinemia and insulin resistance could promote mutually and result in hyperandrogenism, leading to abnormal reproductive function. The pituitary response to gonadotropin-releasing hormone (GnRH) is promoted by hyperinsulinemia, resulting in enhancement of luteinizing hormone and androgen secretion, which, in turn, affects the function of the hypothalamus-pituitary-ovarian gonadal axis (HPO axis) [19]. Hyperandrogenism could inhibit follicle development and follicular atresia and can promote insulin resistance in feedback [20]. Increased pro-inflammatory cytokines and decreased anti-inflammatory factors are related to the pathogenesis of PCOS and this inflammatory condition could damage insulin sensitivity and promote the development of PCOS [21].

In the study, we evaluated the potential protective effects of BAL against PCOS. We revealed that BAL significantly inhibited the development of PCOS, as evidenced by a significant decrease of serum hormone levels, including free testosterone, total testosterone, LH, FSH, progesterone, and estradiol, an improvement of insulin resistance, and an attenuation of functional and pathohistological changes and follicular development, and a decrease of pro-inflammatory cytokines and an increase of anti-inflammatory cytokines in PCOS rats.

It is well-known that AMPK functions as an “energy sensor” in a cell [22]. The dysregulation of AMPK is correlated with both insulin resistance and PCOS endocrine and reproductive function. For example, AMPK could regulate glucose homeostasis and insulin sensitivity through inhibiting inflammation and promoting insulin signaling transduction [23]. In addition, activation of AMPK by metformin and AICAR could attenuate PCOS-related endocrine and reproductive dysfunction [24]. Moreover, AMPK was shown to reduce the activity of NF-κB and the levels of the pro-inflammatory cytokine TNFα [25]. Metformin, the agonist of AMPK, could attenuate TNFα- and chemokine-mediated inflammatory responses and granulocyte dysfunction in an AMPK-dependent manner [26]. Previous studies have suggested that BAL could upregulate AMPK [27] and thus we verified whether activation of AMPK participated in the effects of BAL on PCOS. We used an AMPK inhibitor and showed that inhibition of AMPK could significantly block the inhibitory effects of BAL on PCOS-related endocrine and reproductive dysfunction. The results suggested that AMPK may be a common target of BAL and BAL protected against PCOS in an AMPK-dependent manner.

PI3K/Akt signaling is a major downstream of insulin signaling that mediates the effect of insulin on glucose and lipid metabolism [28–30]. Under the condition of PCOS, PI3K/Akt signaling is usually inhibited [28–30]. In the present study, we evaluated the effect of BAL on PI3K/Akt signaling in PCOS rats. We showed that BAL inhibited the decrease of Akt phosphorylation in an AMPK-dependent manner. The 5α-reductase enzyme is an important metabolic enzyme in the body that regulates androgens [31]. Previous studies have shown that 5α-R1 expression was increased in PCOS skeletal tissues and was negatively correlated with PI3K/Akt signaling [32]. In our study, we found that BAL could decrease the protein level of 5α-R1 in ovarian tissues which effect relied on AMPK. The results suggested, at least partly, AMPK-mediated regulation of PI3K/Akt signaling and 5α-R1 was involved in BAL-induced inhibitory effects on PCOS.

In addition to AMPK/PI3K/Akt signaling, sirtuin 1 (SIRT1) is possibly involved in the BAL-exhibited effects on PCOS. A natural polyphenolic compound, resveratrol, has been shown to promote the expression of SIRT1 in rat ovarian granulosa cells, which implicated the important role of SIRT1 in ovarian function [33]. It has been shown that BAL inhibits the proliferation and migration of human non-small cell lung carcinoma cells through activation of the SIRT1/AMPK signaling pathway [15]. In contrast, Xu et al. showed that BAL inhibited SIRT1-induced regulation of signal transducer and activator of transcription 3 (STAT3), leading to the suppression of excessive hepatic glucose production [34]. Therefore, the role of BAL in animals and humans could be dependent on the characteristics of tissues. Further studies are needed to verify whether SIRT1 is involved in BAL-induced regulation of AMPK signaling and attenuation of PCOS.

There are still several issues to be addressed. Considering that PCOS is a multi-system reproductive metabolic disorder, activation of AMPK signaling may not be the only mechanism responsible for the beneficial effects of BAL [35]. We tried to use dorsomorphin as an AMPK inhibitor. However, it was also reported that dorsomorphin could non-specifically inhibit other kinases such as bone morphogenetic protein (BMP) and VEGF type 2 receptor (FLK1/KDR) signaling [36]. Abnormal BMP [37, 38] and VEGF [36, 39, 40] signalings were reported to be associated with PCOS development. Therefore, we could not exclude the possibility that these signalings were involved in BAL-induced effects on PCOS. Moreover, the influence on hormones and their downstream signaling may also play a role in BAL-exhibited protection against PCOS. For example, BAL could attenuate insulin resistance which effect may contribute to the improvement of PCOS [27, 41, 42]. Furthermore, the DHEA-treated rat is a widely used PCOS model, especially for the study of PCOS with insulin resistance and obesity. However, researchers may encounter frequent aplasia of the ovary or ovarian cyst in maintaining high reproducibility in the induction of PCOS model [43]. This evidence suggests that transgenic mice may be a better animal model to study the exact mechanism of PCOS and the beneficial effects of BAL.

## Conclusions

**Fig. 7:**
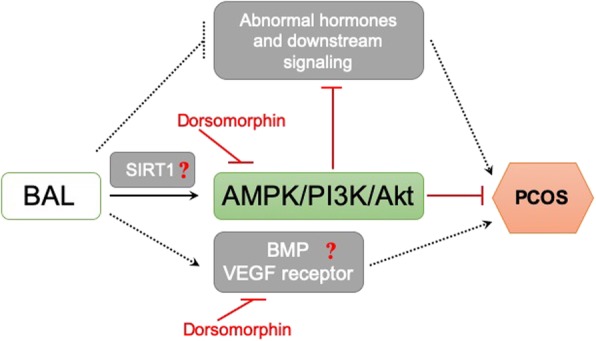
Proposed mechanism of BAL-induced protection against PCOS

In summary, we identified that BAL could attenuate the dysfunction of endocrine and reproduction in PCOS rats. BAL ameliorated the complications of PCOS, including abnormal secretion of hormones, insulin resistance, pathohistological changes, and inflammation. Among those mechanisms, the upregulation of AMPK contributed to the beneficial effect of BAL (Fig. 7). The results highlight the potential role of BAL for the intervention of PCOS.

## Data Availability

All the data is contained in the manuscript.

## Abbreviations

5α-R1: 5α-reductase type 1
AMPK: AMP-activated protein kinase
BAL: Baicalin
BMP: Bone morphogenetic protein
DHEA: Dehydroepiandrosterone
E2: Estradiol
FSH: Follicle-stimulating hormone
HOMA-IR: Insulin resistance index
LH: Luteinizing hormone
P: Progesterone
PCOS: Polycystic ovary syndrome
SIRT1: Sirtuin 1
